# *N*,*N*′-Bis(4-chloro­benzyl­idene)-3,3′-dimeth­oxy­biphenyl-4,4′-diamine

**DOI:** 10.1107/S1600536811015467

**Published:** 2011-05-07

**Authors:** Ashokkumar Subashini, Kandasamy Ramamurthi, Helen Stoeckli-Evans

**Affiliations:** a Crystal Growth and Thin Film Laboratory, School of Physics, Bharathidasan, University, Tiruchirappalli, Tamil Nadu 620 024, India; b Institute of Physics, University of Neuchâtel, Rue Emile-Argand 11, CH-2000 Neuchâtel, Switzerland

## Abstract

The title compound, C_28_H_22_Cl_2_N_2_O_2_, crystallized with two independent mol­ecules (*A* and *B*) in the asymmetric unit. The two mol­ecules differ essentially in the orientation of the outer aromatic rings. These dihedral angles are 56.07 (13) and 27.62 (15) Å for mol­ecules *A* and *B*, respectively. In the crystal, *A* mol­ecules are related as centrosymmetric pairs through a weak π–π inter­action [centroid–centroid distance = 3.6959 (15) Å]. There are also a number of inter­molecular C—H⋯O, C—H⋯N and C—H⋯π inter­actions present.

## Related literature

For early work on the synthesis of multidentate Schiff base ligands, see: Weber (1967 ▶); Lesser *et al.* (1975 ▶); Munro & Camp (2003 ▶). For examples of Schiff base metal complexes exhibiting biological properties, see: Golcu *et al.* (2005 ▶); Liu & Yang (2010 ▶). For examples of Schiff base metal complexes exhibiting catalytic properties, see: Daier *et al.* (2004 ▶). For details of photochromic properties of some Schiff base complexes, see: Zgierski & Grabowska (2000 ▶). For examples of some similar 4,4′-biphenyl diamine Schiff bases, see: Lesser *et al.* (1975 ▶); Aygun *et al.* (2004 ▶); Hou *et al.* (2006 ▶). For details of the Cambridge Structural Database, see: Allen (2002 ▶).
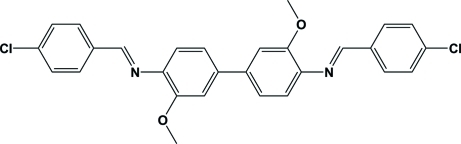


## Experimental

### 

#### Crystal data

C_28_H_22_Cl_2_N_2_O_2_
*M* *_r_* = 489.38
Triclinic, 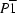

*a* = 9.2358 (5) Å
*b* = 11.8559 (6) Å
*c* = 23.5218 (12) Å
α = 75.810 (4)°
β = 80.326 (4)°
γ = 79.288 (4)°
*V* = 2433.1 (2) Å^3^
*Z* = 4
Mo *K*α radiation
μ = 0.30 mm^−1^
*T* = 173 K
0.45 × 0.18 × 0.12 mm

#### Data collection

Stoe IPDS 2 diffractometer
Absorption correction: multi-scan (*MULscanABS* in *PLATON*; Spek, 2009 ▶) *T*_min_ = 0.772, *T*_max_ = 1.000
29251 measured reflections
9183 independent reflections
5705 reflections with *I* > 2σ(*I*)
*R*_int_ = 0.071

#### Refinement

*R*[*F* ^2^ > 2σ(*F* ^2^)] = 0.053
*wR*(*F* ^2^) = 0.125
*S* = 0.98
9183 reflections
617 parameters
H-atom parameters constrained
Δρ_max_ = 0.26 e Å^−3^
Δρ_min_ = −0.30 e Å^−3^

### 

Data collection: *X-AREA* (Stoe & Cie, 2009 ▶); cell refinement: *X-AREA*; data reduction: *X-RED32* (Stoe & Cie, 2009 ▶); program(s) used to solve structure: *SHELXS97* (Sheldrick, 2008 ▶); program(s) used to refine structure: *SHELXL97* (Sheldrick, 2008 ▶); molecular graphics: *PLATON* (Spek, 2009 ▶) and *Mercury* (Macrae *et al.*, 2006 ▶); software used to prepare material for publication: *SHELXL97*, *PLATON* and *publCIF* (Westrip, 2010 ▶).

## Supplementary Material

Crystal structure: contains datablocks I, global. DOI: 10.1107/S1600536811015467/fl2345sup1.cif

Structure factors: contains datablocks I. DOI: 10.1107/S1600536811015467/fl2345Isup2.hkl

Supplementary material file. DOI: 10.1107/S1600536811015467/fl2345Isup3.cml

Additional supplementary materials:  crystallographic information; 3D view; checkCIF report

## Figures and Tables

**Table 1:** Hydrogen-bond geometry (Å, °) *Cg*2, *Cg*5 and *Cg*7 are the centroids of the C8–C13, C29–C34 and C42–C47 rings, respectively.

*D*—H⋯*A*	*D*—H	H⋯*A*	*D*⋯*A*	*D*—H⋯*A*
C23—H23⋯O4^i^	0.95	2.48	3.348 (4)	152
C31—H31⋯N1^ii^	0.95	2.57	3.498 (4)	166
C3—H3⋯*Cg*5^iii^	0.95	2.75	3.642 (3)	156
C30—H30⋯*Cg*2^ii^	0.95	2.90	3.641 (3)	136
C33—H33⋯*Cg*7^iv^	0.95	2.97	3.850 (3)	154

Symmetry codes: (i) 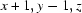; (ii) 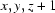; (iii) 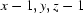; (iv) 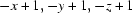.

**Fig. 1.**
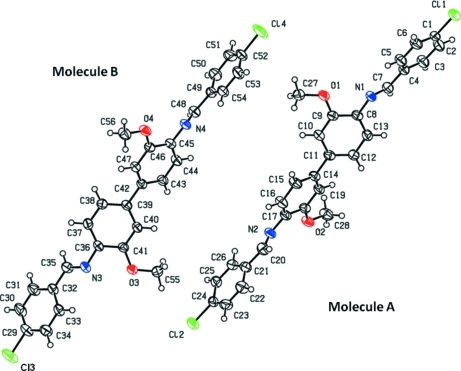
A view of the molecular structure of the two independent molecules (A and B) of the title compound with the atom numbering. The displacement ellipsoids are drawn at the 50% probability level. Hydrogen atoms are shown as spheres of arbitrary radii.

**Fig. 2.**
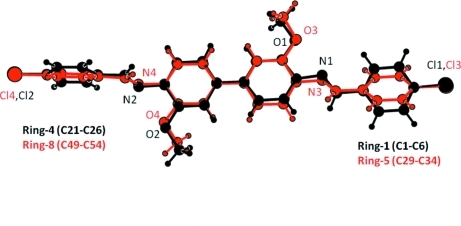
A view of the auto-fit of molecule B (red) inverted on molecule A (black); the best weighted and unit weight RMS-fit parameters are 0.843 and 1.065 Å, for 34 fitted atoms [Auto-fit routine in PLATON (Spek, 2009)].

**Fig. 3.**
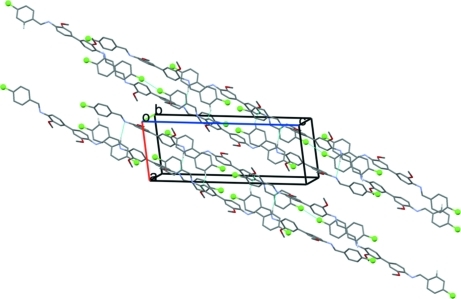
A view along the b axis of the crystal packing of the title compound. The intermolecular C–H..O and C–H..N interactions, and the short Cl1···Cl2i interactions [3.5048 (12) Å; symmetry code (i) = x - 1, x + 2, x - 1], are shown as dashed cyan lines. H-atoms not involved in these interactions have been omitted for clarity.

## supplementary crystallographic information

### Comment

Multidentate Schiff base ligands have been studied for many years (Weber, 1967; Lesser *et al.*, 1975; Munro & Camp, 2003). Metal complexes of multidentate Schiff base ligands play an important role in the development of coordination chemistry and possess important properties, such as biological activity (Golcu *et al.*, 2005; Liu & Yang, 2010), catalytic activity (Daier *et al.*, 2004) and photochromic properties (Zgierski & Grabowska, 2000). Because of the structural characteristics of the Schiff base products (*i.e.* electron donor and acceptor groups connected to a π-conjugated chain), they will have potential as NLO or electro-optical materials. We present here the synthesis and crystal structure of the title 4,4'-biphenyl diamine Schiff base compound, prepared by the condensation reaction of 3,3'-dimethoxybenzidine with two equivalents of 4-chlorobenzaldehyde. There are a number of examples of similar Schiff bases in the Cambridge Structural Database (Allen, 2002); for example, 2,2'-Dibromo-4,4'-bis(*p*-methoxybenzilideneamine)biphenyl (Lesser *et al.*, 1975), 3,3'Dimethoxybenzidene(2-hydroxybnzilidene)amino)biphenyl (Aygun *et al.*, 2004) and 4,4'-bis(2,6-Dichlorobenzilideneamino)biphenyl (Hou *et al.*, 2006).

The title compound crystallized with two independent molecules (A and B) in the asymmetric unit, Fig. 1. The 3,3'-dimethoxybiphenyl moieties in the two molecules have similar conformations, with the aromatic rings being inclined to one another by 36.12 (13) ° in molecule A and by 38.09 (13) ° in molecule B. A view of the Auto-fit   figure (Fig. 2; Spek, 2009) of inverted molecule B on molecule A illustrates the difference in the conformation of the two molecules. The outer aromatic rings, Ring-1 (C1—C6) and Ring-4 (C21—C26), are inclined to one another by 56.05 (13) ° in molecule A, while in molecule B Ring-5 (C29—C34) and Ring-8 (49-C54) are inclined to one another by 27.62 (15) °. In molecule A Ring-1 (C1—C6) is inclined to Ring-2 (C14—C19) and Ring-3 (C21—C26) by 10.26 (13) and 56.07 (13) °, respectively. This is different to the situation in molecule B where Ring-5 (C29—C34) is inclined to Ring-7 (C42—C47) and Ring-8 (C49—C54) by 85.70 (13) and 27.62 (15) °, respectively.

In the crystal the A molecules stack head-to-tail with a separation of the Cl atoms, Cl1···Cl2^i^, of 3.5048 (12) Å (symmetry code (i) = *x* - 1, *x* + 2, *x* - 1). The B molecules stack head-to-head with a shorter separation of the Cl atoms; the distance Cl4···Cl4^ii^ being 3.3173 (18) Å (symmetry code (ii) = -*x* - 1, -*y* + 3, -*z*). The crystal structure is further stabilized by intermolecular C–H···O and C–H···N hydrogen bonds (Table 1 and Fig. 3). The A molecules are related as centrosymmetric pairs through a weak π–π interaction [*Cg*2···*Cg*2^i^ = 3.6959 (15) Å; *Cg*2 is the centroid of Ring-2 (C8—C13); Symmetry code: (i) -*x*, 1 - *y*, -*z*].

Footnote to Table 1: *Cg*2 is the centroid of Ring-2 (C8—C13); *Cg*5 is the centroid of Ring-5 (C29—C34); *Cg*7 is the centroid of Ring-7 (C42—C47).

### Experimental

The title compound was synthesized by mixing 3,3'-dimethoxybenzidine and 4-chlorobenzaldehyde (molar ratio 1:2) in methanol at 343 K for 1 h. The mixture was then allowed to cool to RT giving a brown solid. Recrystallization from methanol gave brown rod-shaped crystals of the title compound suitable for X-ray diffraction analysis.

### Refinement

The C-bound H-atoms were included in calculated positions and treated as riding atoms: C—H = 0.95 and 0.98 Å for CH(aromatic) and CH_3_, respectively, with *U*_iso_(H) = k × *U*_eq_(parent C-atom), where k = 1.5 for CH_3_ H-atoms and k = 1.2 for all other H-atoms.

### Crystal data

**Table:**

C_28_H_22_Cl_2_N_2_O_2_	*Z* = 4
*M**_r_* = 489.38	*F*(000) = 1016
Triclinic, *P*1	*D*_x_ = 1.336 Mg m^−^^3^
Hall symbol: -P 1	Mo *K*α radiation, λ = 0.71073 Å
*a* = 9.2358 (5) Å	Cell parameters from 16478 reflections
*b* = 11.8559 (6) Å	θ = 1.8–26.0°
*c* = 23.5218 (12) Å	µ = 0.30 mm^−^^1^
α = 75.810 (4)°	*T* = 173 K
β = 80.326 (4)°	Rod, brown
γ = 79.288 (4)°	0.45 × 0.18 × 0.12 mm
*V* = 2433.1 (2) Å^3^	

### Data collection

**Table:**

Stoe IPDS 2 diffractometer	9183 independent reflections
Radiation source: fine-focus sealed tube	5705 reflections with *I* > 2σ(*I*)
graphite	*R*_int_ = 0.071
φ and ω scans	θ_max_ = 25.6°, θ_min_ = 1.8°
Absorption correction: multi-scan (MULscanABS in *PLATON*; Spek, 2009)	*h* = −11→11
*T*_min_ = 0.772, *T*_max_ = 1.000	*k* = −14→14
29251 measured reflections	*l* = −28→28

### Refinement

**Table:**

Refinement on *F*^2^	Primary atom site location: structure-invariant direct methods
Least-squares matrix: full	Secondary atom site location: difference Fourier map
*R*[*F*^2^ > 2σ(*F*^2^)] = 0.053	Hydrogen site location: inferred from neighbouring sites
*wR*(*F*^2^) = 0.125	H-atom parameters constrained
*S* = 0.98	*w* = 1/[σ^2^(*F*_o_^2^) + (0.0607*P*)^2^] where *P* = (*F*_o_^2^ + 2*F*_c_^2^)/3
9183 reflections	(Δ/σ)_max_ = 0.001
617 parameters	Δρ_max_ = 0.26 e Å^−^^3^
0 restraints	Δρ_min_ = −0.30 e Å^−^^3^

### Special details

**Table:**

Geometry. Bond distances, angles *etc*. have been calculated using the rounded fractional coordinates. All su's are estimated from the variances of the (full) variance-covariance matrix. The cell e.s.d.'s are taken into account in the estimation of distances, angles and torsion angles

### Fractional atomic coordinates and isotropic or equivalent isotropic displacement parameters (Å^2^)

**Table:**

	*x*	*y*	*z*	*U*_iso_*/*U*_eq_	
Cl1	−0.16693 (10)	0.73278 (7)	−0.42605 (3)	0.0557 (3)	
Cl2	0.76750 (12)	0.00726 (7)	0.47655 (4)	0.0700 (3)	
O1	0.1449 (2)	0.70175 (15)	−0.09211 (8)	0.0440 (7)	
O2	0.2304 (2)	0.10960 (15)	0.19381 (8)	0.0423 (6)	
N1	0.0777 (3)	0.57226 (18)	−0.15956 (9)	0.0379 (7)	
N2	0.4364 (3)	0.19921 (19)	0.22848 (9)	0.0399 (8)	
C1	−0.1290 (3)	0.6770 (2)	−0.35318 (11)	0.0402 (9)	
C2	−0.2099 (4)	0.5962 (3)	−0.31557 (13)	0.0497 (11)	
C3	−0.1785 (3)	0.5547 (3)	−0.25790 (13)	0.0478 (10)	
C4	−0.0646 (3)	0.5919 (2)	−0.23855 (11)	0.0393 (9)	
C5	0.0142 (3)	0.6737 (2)	−0.27775 (12)	0.0461 (10)	
C6	−0.0169 (3)	0.7171 (2)	−0.33514 (12)	0.0473 (10)	
C7	−0.0298 (3)	0.5443 (2)	−0.17770 (12)	0.0425 (9)	
C8	0.1164 (3)	0.5157 (2)	−0.10280 (11)	0.0348 (8)	
C9	0.1611 (3)	0.5830 (2)	−0.06896 (11)	0.0352 (8)	
C10	0.2146 (3)	0.5283 (2)	−0.01568 (11)	0.0361 (9)	
C11	0.2278 (3)	0.4060 (2)	0.00542 (11)	0.0352 (8)	
C12	0.1835 (3)	0.3401 (2)	−0.02828 (11)	0.0395 (9)	
C13	0.1285 (3)	0.3946 (2)	−0.08133 (11)	0.0398 (9)	
C14	0.2887 (3)	0.3500 (2)	0.06174 (11)	0.0352 (8)	
C15	0.4010 (3)	0.3933 (2)	0.07895 (12)	0.0447 (9)	
C16	0.4541 (3)	0.3410 (2)	0.13245 (12)	0.0454 (10)	
C17	0.3966 (3)	0.2461 (2)	0.17100 (11)	0.0378 (9)	
C18	0.2828 (3)	0.2025 (2)	0.15388 (11)	0.0359 (8)	
C19	0.2320 (3)	0.2536 (2)	0.09986 (11)	0.0359 (8)	
C20	0.5723 (4)	0.1773 (2)	0.23557 (12)	0.0470 (10)	
C21	0.6195 (3)	0.1368 (2)	0.29511 (12)	0.0409 (9)	
C22	0.7661 (3)	0.0873 (3)	0.30185 (14)	0.0519 (11)	
C23	0.8113 (4)	0.0469 (2)	0.35752 (15)	0.0529 (11)	
C24	0.7101 (4)	0.0574 (2)	0.40639 (13)	0.0466 (10)	
C25	0.5640 (3)	0.1055 (2)	0.40128 (12)	0.0449 (10)	
C26	0.5199 (3)	0.1443 (2)	0.34562 (12)	0.0417 (9)	
C27	0.1841 (4)	0.7738 (2)	−0.05816 (14)	0.0552 (10)	
C28	0.1111 (3)	0.0658 (3)	0.17929 (13)	0.0509 (10)	
Cl3	0.72546 (11)	0.22366 (9)	0.91179 (4)	0.0780 (4)	
Cl4	−0.40375 (15)	0.99270 (9)	0.05465 (5)	0.1072 (5)	
O3	0.4123 (2)	0.30306 (14)	0.58097 (8)	0.0405 (6)	
O4	0.1571 (2)	0.91381 (15)	0.32380 (8)	0.0416 (6)	
N3	0.4599 (3)	0.43315 (19)	0.65042 (9)	0.0392 (8)	
N4	−0.0211 (3)	0.79885 (18)	0.28786 (9)	0.0371 (7)	
C29	0.6336 (3)	0.2908 (3)	0.85004 (12)	0.0470 (10)	
C30	0.5040 (3)	0.3671 (3)	0.85675 (12)	0.0533 (11)	
C31	0.4329 (3)	0.4206 (3)	0.80728 (12)	0.0509 (10)	
C32	0.4907 (3)	0.3992 (2)	0.75239 (11)	0.0373 (9)	
C33	0.6227 (3)	0.3212 (2)	0.74676 (12)	0.0411 (9)	
C34	0.6943 (3)	0.2663 (3)	0.79566 (13)	0.0490 (10)	
C35	0.4114 (3)	0.4551 (2)	0.70078 (12)	0.0413 (9)	
C36	0.3826 (3)	0.4902 (2)	0.60174 (10)	0.0338 (8)	
C37	0.3332 (3)	0.6105 (2)	0.58768 (11)	0.0415 (9)	
C38	0.2646 (3)	0.6615 (2)	0.53740 (11)	0.0410 (9)	
C39	0.2441 (3)	0.5941 (2)	0.49943 (10)	0.0330 (8)	
C40	0.2939 (3)	0.4727 (2)	0.51341 (10)	0.0331 (8)	
C41	0.3628 (3)	0.4211 (2)	0.56359 (11)	0.0329 (8)	
C42	0.1699 (3)	0.6476 (2)	0.44576 (10)	0.0324 (8)	
C43	0.0753 (3)	0.5893 (2)	0.42723 (11)	0.0371 (8)	
C44	0.0071 (3)	0.6402 (2)	0.37685 (11)	0.0363 (8)	
C45	0.0340 (3)	0.7494 (2)	0.34255 (10)	0.0333 (8)	
C46	0.1313 (3)	0.8083 (2)	0.36068 (11)	0.0332 (8)	
C47	0.1964 (3)	0.7585 (2)	0.41185 (10)	0.0333 (8)	
C48	−0.1580 (3)	0.7995 (2)	0.28454 (12)	0.0419 (9)	
C49	−0.2182 (3)	0.8436 (2)	0.22789 (13)	0.0410 (9)	
C50	−0.3700 (4)	0.8773 (3)	0.22698 (16)	0.0584 (12)	
C51	−0.4283 (4)	0.9242 (3)	0.17360 (19)	0.0694 (14)	
C52	−0.3328 (5)	0.9342 (3)	0.12174 (17)	0.0634 (13)	
C53	−0.1824 (4)	0.8986 (3)	0.12114 (14)	0.0577 (13)	
C54	−0.1256 (4)	0.8540 (2)	0.17442 (12)	0.0464 (10)	
C55	0.3890 (4)	0.2297 (2)	0.54500 (13)	0.0507 (10)	
C56	0.2591 (4)	0.9755 (2)	0.33901 (13)	0.0493 (10)	
H2	−0.28600	0.56920	−0.32890	0.0600*	
H3	−0.23530	0.50020	−0.23120	0.0570*	
H5	0.09120	0.70050	−0.26490	0.0550*	
H6	0.03760	0.77340	−0.36170	0.0570*	
H7	−0.08930	0.49140	−0.15130	0.0510*	
H10	0.24300	0.57480	0.00700	0.0430*	
H12	0.19100	0.25710	−0.01490	0.0470*	
H13	0.09830	0.34790	−0.10350	0.0480*	
H15	0.44130	0.45910	0.05390	0.0540*	
H16	0.53190	0.37090	0.14310	0.0540*	
H19	0.15660	0.22220	0.08850	0.0430*	
H20	0.64560	0.18710	0.20190	0.0570*	
H22	0.83620	0.08100	0.26780	0.0620*	
H23	0.91140	0.01230	0.36170	0.0630*	
H25	0.49460	0.11180	0.43550	0.0540*	
H26	0.41900	0.17690	0.34190	0.0500*	
H27A	0.28880	0.74970	−0.05210	0.0830*	
H27B	0.12170	0.76480	−0.01980	0.0830*	
H27C	0.16880	0.85640	−0.07940	0.0830*	
H28A	0.14360	0.03600	0.14310	0.0760*	
H28B	0.02690	0.12930	0.17300	0.0760*	
H28C	0.08070	0.00190	0.21180	0.0760*	
H30	0.46380	0.38300	0.89440	0.0640*	
H31	0.34250	0.47300	0.81140	0.0610*	
H33	0.66370	0.30580	0.70910	0.0490*	
H34	0.78360	0.21250	0.79200	0.0590*	
H35	0.32230	0.50870	0.70540	0.0500*	
H37	0.34660	0.65840	0.61280	0.0500*	
H38	0.23110	0.74380	0.52870	0.0490*	
H40	0.28020	0.42500	0.48820	0.0400*	
H43	0.05700	0.51360	0.44940	0.0450*	
H44	−0.05920	0.59960	0.36560	0.0440*	
H47	0.25980	0.80010	0.42410	0.0400*	
H48	−0.22170	0.77090	0.31920	0.0500*	
H50	−0.43510	0.86850	0.26310	0.0700*	
H51	−0.53210	0.94880	0.17320	0.0830*	
H53	−0.11830	0.90450	0.08480	0.0690*	
H54	−0.02160	0.83010	0.17440	0.0560*	
H55A	0.43770	0.25550	0.50470	0.0760*	
H55B	0.28220	0.23480	0.54430	0.0760*	
H55C	0.43100	0.14810	0.56120	0.0760*	
H56A	0.35490	0.92420	0.34290	0.0740*	
H56B	0.21950	0.99830	0.37660	0.0740*	
H56C	0.27280	1.04610	0.30790	0.0740*	

### Atomic displacement parameters (Å^2^)

**Table:**

	*U*^11^	*U*^22^	*U*^33^	*U*^12^	*U*^13^	*U*^23^
Cl1	0.0751 (6)	0.0561 (4)	0.0355 (4)	−0.0003 (4)	−0.0251 (4)	−0.0043 (3)
Cl2	0.1128 (8)	0.0452 (4)	0.0613 (5)	−0.0069 (4)	−0.0571 (5)	−0.0019 (4)
O1	0.0574 (13)	0.0350 (10)	0.0411 (11)	−0.0066 (9)	−0.0244 (10)	0.0007 (8)
O2	0.0463 (12)	0.0427 (10)	0.0345 (10)	−0.0121 (9)	−0.0138 (9)	0.0079 (8)
N1	0.0447 (14)	0.0417 (12)	0.0265 (11)	−0.0009 (10)	−0.0120 (10)	−0.0053 (9)
N2	0.0458 (15)	0.0419 (13)	0.0308 (12)	−0.0064 (11)	−0.0136 (10)	0.0001 (9)
C1	0.0523 (18)	0.0380 (15)	0.0290 (14)	0.0033 (13)	−0.0152 (13)	−0.0058 (11)
C2	0.056 (2)	0.0555 (18)	0.0438 (17)	−0.0151 (15)	−0.0209 (15)	−0.0079 (14)
C3	0.0536 (19)	0.0512 (17)	0.0400 (16)	−0.0161 (14)	−0.0134 (14)	−0.0018 (13)
C4	0.0450 (17)	0.0403 (15)	0.0322 (14)	−0.0010 (13)	−0.0106 (12)	−0.0074 (12)
C5	0.0530 (19)	0.0478 (16)	0.0387 (16)	−0.0128 (14)	−0.0154 (14)	−0.0015 (13)
C6	0.0537 (19)	0.0493 (17)	0.0371 (15)	−0.0098 (14)	−0.0142 (14)	0.0010 (13)
C7	0.0498 (19)	0.0425 (15)	0.0345 (15)	−0.0039 (13)	−0.0087 (13)	−0.0071 (12)
C8	0.0323 (15)	0.0419 (15)	0.0265 (13)	−0.0035 (11)	−0.0034 (11)	−0.0024 (11)
C9	0.0360 (16)	0.0360 (14)	0.0313 (14)	−0.0024 (11)	−0.0082 (12)	−0.0030 (11)
C10	0.0366 (16)	0.0421 (15)	0.0297 (14)	−0.0057 (12)	−0.0096 (12)	−0.0045 (11)
C11	0.0342 (15)	0.0410 (15)	0.0254 (13)	−0.0025 (12)	−0.0023 (11)	−0.0012 (11)
C12	0.0502 (18)	0.0356 (14)	0.0306 (14)	−0.0043 (12)	−0.0080 (13)	−0.0030 (11)
C13	0.0477 (18)	0.0420 (15)	0.0313 (14)	−0.0075 (13)	−0.0096 (13)	−0.0073 (12)
C14	0.0337 (15)	0.0418 (15)	0.0271 (13)	−0.0016 (12)	−0.0061 (11)	−0.0035 (11)
C15	0.0465 (18)	0.0499 (16)	0.0339 (15)	−0.0136 (14)	−0.0115 (13)	0.0068 (12)
C16	0.0463 (18)	0.0521 (17)	0.0379 (15)	−0.0154 (14)	−0.0150 (13)	0.0024 (13)
C17	0.0418 (17)	0.0409 (15)	0.0283 (13)	−0.0030 (13)	−0.0102 (12)	−0.0020 (11)
C18	0.0374 (16)	0.0362 (14)	0.0305 (14)	−0.0039 (12)	−0.0046 (12)	−0.0020 (11)
C19	0.0385 (16)	0.0404 (15)	0.0274 (13)	−0.0037 (12)	−0.0072 (12)	−0.0044 (11)
C20	0.053 (2)	0.0515 (17)	0.0338 (15)	−0.0104 (15)	−0.0049 (14)	−0.0029 (13)
C21	0.0422 (17)	0.0395 (15)	0.0409 (16)	−0.0059 (13)	−0.0133 (13)	−0.0034 (12)
C22	0.0463 (19)	0.0580 (18)	0.0496 (18)	−0.0049 (15)	−0.0097 (15)	−0.0082 (15)
C23	0.0469 (19)	0.0454 (17)	0.068 (2)	0.0007 (14)	−0.0264 (17)	−0.0087 (15)
C24	0.067 (2)	0.0296 (14)	0.0500 (18)	−0.0074 (14)	−0.0317 (16)	−0.0055 (12)
C25	0.060 (2)	0.0396 (15)	0.0369 (15)	−0.0075 (14)	−0.0129 (14)	−0.0074 (12)
C26	0.0441 (17)	0.0384 (15)	0.0421 (16)	−0.0027 (13)	−0.0133 (14)	−0.0056 (12)
C27	0.076 (2)	0.0361 (15)	0.0582 (19)	−0.0058 (15)	−0.0318 (17)	−0.0055 (14)
C28	0.0522 (19)	0.0506 (17)	0.0459 (17)	−0.0179 (15)	−0.0138 (15)	0.0093 (14)
Cl3	0.0771 (7)	0.1085 (7)	0.0427 (5)	0.0008 (5)	−0.0327 (4)	−0.0004 (4)
Cl4	0.1606 (12)	0.0747 (6)	0.1045 (8)	−0.0176 (7)	−0.1093 (9)	0.0083 (6)
O3	0.0538 (12)	0.0333 (9)	0.0357 (10)	−0.0049 (8)	−0.0204 (9)	−0.0017 (8)
O4	0.0491 (12)	0.0377 (10)	0.0373 (10)	−0.0147 (9)	−0.0171 (9)	0.0067 (8)
N3	0.0441 (14)	0.0452 (13)	0.0307 (12)	−0.0057 (11)	−0.0137 (10)	−0.0077 (10)
N4	0.0399 (14)	0.0415 (12)	0.0297 (12)	−0.0051 (10)	−0.0137 (10)	−0.0020 (9)
C29	0.0462 (19)	0.0621 (19)	0.0322 (15)	−0.0102 (15)	−0.0160 (13)	−0.0007 (13)
C30	0.053 (2)	0.078 (2)	0.0290 (15)	−0.0047 (17)	−0.0087 (14)	−0.0137 (14)
C31	0.0435 (18)	0.070 (2)	0.0372 (16)	0.0045 (15)	−0.0077 (13)	−0.0160 (14)
C32	0.0368 (16)	0.0468 (16)	0.0291 (14)	−0.0058 (13)	−0.0075 (12)	−0.0080 (11)
C33	0.0407 (17)	0.0527 (16)	0.0313 (14)	−0.0064 (13)	−0.0069 (12)	−0.0107 (12)
C34	0.0433 (18)	0.0587 (18)	0.0442 (17)	−0.0001 (14)	−0.0134 (14)	−0.0102 (14)
C35	0.0384 (16)	0.0477 (16)	0.0358 (15)	−0.0021 (13)	−0.0077 (12)	−0.0066 (12)
C36	0.0362 (15)	0.0407 (14)	0.0251 (13)	−0.0085 (12)	−0.0086 (11)	−0.0033 (11)
C37	0.0567 (19)	0.0382 (15)	0.0330 (14)	−0.0079 (13)	−0.0136 (13)	−0.0087 (11)
C38	0.0583 (19)	0.0315 (13)	0.0331 (14)	−0.0056 (13)	−0.0140 (13)	−0.0026 (11)
C39	0.0347 (15)	0.0357 (14)	0.0275 (13)	−0.0070 (11)	−0.0066 (11)	−0.0018 (11)
C40	0.0367 (15)	0.0358 (14)	0.0280 (13)	−0.0096 (11)	−0.0082 (11)	−0.0035 (11)
C41	0.0357 (15)	0.0314 (13)	0.0304 (13)	−0.0061 (11)	−0.0080 (11)	−0.0011 (10)
C42	0.0327 (15)	0.0369 (14)	0.0265 (13)	−0.0039 (11)	−0.0049 (11)	−0.0055 (11)
C43	0.0419 (16)	0.0361 (14)	0.0324 (14)	−0.0082 (12)	−0.0089 (12)	−0.0013 (11)
C44	0.0347 (15)	0.0431 (15)	0.0324 (14)	−0.0119 (12)	−0.0095 (12)	−0.0026 (11)
C45	0.0298 (15)	0.0396 (14)	0.0289 (13)	−0.0028 (11)	−0.0071 (11)	−0.0044 (11)
C46	0.0328 (15)	0.0361 (14)	0.0287 (13)	−0.0053 (11)	−0.0041 (11)	−0.0030 (11)
C47	0.0341 (15)	0.0365 (14)	0.0299 (13)	−0.0067 (11)	−0.0077 (11)	−0.0052 (11)
C48	0.0429 (18)	0.0457 (16)	0.0367 (15)	−0.0068 (13)	−0.0102 (13)	−0.0050 (12)
C49	0.0414 (17)	0.0377 (15)	0.0481 (17)	−0.0052 (12)	−0.0200 (14)	−0.0085 (12)
C50	0.047 (2)	0.062 (2)	0.073 (2)	−0.0036 (16)	−0.0259 (17)	−0.0189 (17)
C51	0.060 (2)	0.057 (2)	0.102 (3)	0.0044 (17)	−0.052 (2)	−0.020 (2)
C52	0.090 (3)	0.0412 (17)	0.072 (2)	−0.0083 (17)	−0.060 (2)	−0.0046 (16)
C53	0.084 (3)	0.0481 (17)	0.0472 (18)	−0.0153 (17)	−0.0320 (18)	−0.0025 (14)
C54	0.0548 (19)	0.0441 (16)	0.0435 (17)	−0.0075 (14)	−0.0236 (15)	−0.0042 (13)
C55	0.074 (2)	0.0327 (15)	0.0489 (17)	−0.0053 (14)	−0.0258 (16)	−0.0059 (13)
C56	0.060 (2)	0.0463 (16)	0.0423 (16)	−0.0218 (15)	−0.0163 (14)	0.0056 (13)

### Geometric parameters (Å, °)

**Table:**

Cl1—C1	1.749 (3)	C22—H22	0.9500
Cl2—C24	1.748 (3)	C23—H23	0.9500
Cl3—C29	1.749 (3)	C25—H25	0.9500
Cl4—C52	1.744 (4)	C26—H26	0.9500
O1—C9	1.369 (3)	C27—H27C	0.9800
O1—C27	1.430 (3)	C27—H27B	0.9800
O2—C28	1.424 (4)	C27—H27A	0.9800
O2—C18	1.367 (3)	C28—H28A	0.9800
O3—C41	1.371 (3)	C28—H28B	0.9800
O3—C55	1.418 (3)	C28—H28C	0.9800
O4—C46	1.372 (3)	C29—C30	1.371 (5)
O4—C56	1.431 (4)	C29—C34	1.384 (4)
N1—C7	1.270 (4)	C30—C31	1.388 (4)
N1—C8	1.412 (3)	C31—C32	1.377 (4)
N2—C17	1.412 (3)	C32—C33	1.395 (4)
N2—C20	1.264 (5)	C32—C35	1.474 (4)
N3—C35	1.265 (3)	C33—C34	1.383 (4)
N3—C36	1.412 (3)	C36—C37	1.389 (3)
N4—C45	1.413 (3)	C36—C41	1.408 (3)
N4—C48	1.278 (4)	C37—C38	1.387 (4)
C1—C2	1.374 (4)	C38—C39	1.391 (3)
C1—C6	1.381 (4)	C39—C40	1.401 (3)
C2—C3	1.385 (4)	C39—C42	1.484 (3)
C3—C4	1.393 (4)	C40—C41	1.388 (4)
C4—C7	1.470 (4)	C42—C43	1.390 (4)
C4—C5	1.385 (4)	C42—C47	1.402 (3)
C5—C6	1.380 (4)	C43—C44	1.387 (4)
C8—C9	1.409 (4)	C44—C45	1.389 (3)
C8—C13	1.390 (3)	C45—C46	1.409 (4)
C9—C10	1.388 (4)	C46—C47	1.386 (4)
C10—C11	1.402 (3)	C48—C49	1.469 (4)
C11—C14	1.481 (4)	C49—C50	1.387 (5)
C11—C12	1.392 (4)	C49—C54	1.389 (4)
C12—C13	1.389 (4)	C50—C51	1.396 (6)
C14—C15	1.394 (4)	C51—C52	1.374 (6)
C14—C19	1.395 (4)	C52—C53	1.373 (6)
C15—C16	1.386 (4)	C53—C54	1.385 (4)
C16—C17	1.389 (4)	C30—H30	0.9500
C17—C18	1.407 (4)	C31—H31	0.9500
C18—C19	1.385 (4)	C33—H33	0.9500
C20—C21	1.476 (4)	C34—H34	0.9500
C21—C22	1.391 (4)	C35—H35	0.9500
C21—C26	1.386 (4)	C37—H37	0.9500
C22—C23	1.386 (5)	C38—H38	0.9500
C23—C24	1.370 (5)	C40—H40	0.9500
C24—C25	1.377 (5)	C43—H43	0.9500
C25—C26	1.381 (4)	C44—H44	0.9500
C2—H2	0.9500	C47—H47	0.9500
C3—H3	0.9500	C48—H48	0.9500
C5—H5	0.9500	C50—H50	0.9500
C6—H6	0.9500	C51—H51	0.9500
C7—H7	0.9500	C53—H53	0.9500
C10—H10	0.9500	C54—H54	0.9500
C12—H12	0.9500	C55—H55A	0.9800
C13—H13	0.9500	C55—H55B	0.9800
C15—H15	0.9500	C55—H55C	0.9800
C16—H16	0.9500	C56—H56A	0.9800
C19—H19	0.9500	C56—H56B	0.9800
C20—H20	0.9500	C56—H56C	0.9800
			
Cl1···Cl2^i^	3.5048 (12)	Cl4···Cl4^ii^	3.3173 (18)
			
C9—O1—C27	117.1 (2)	H28A—C28—H28B	110.00
C18—O2—C28	117.0 (2)	O2—C28—H28A	109.00
C41—O3—C55	116.9 (2)	O2—C28—H28B	109.00
C46—O4—C56	117.7 (2)	O2—C28—H28C	109.00
C7—N1—C8	119.7 (2)	Cl3—C29—C30	119.1 (2)
C17—N2—C20	119.2 (2)	Cl3—C29—C34	119.2 (2)
C35—N3—C36	119.7 (2)	C30—C29—C34	121.8 (3)
C45—N4—C48	119.0 (2)	C29—C30—C31	118.5 (3)
Cl1—C1—C6	118.0 (2)	C30—C31—C32	121.4 (3)
C2—C1—C6	121.8 (3)	C31—C32—C33	119.0 (3)
Cl1—C1—C2	120.2 (2)	C31—C32—C35	120.3 (2)
C1—C2—C3	118.9 (3)	C33—C32—C35	120.6 (2)
C2—C3—C4	120.8 (3)	C32—C33—C34	120.4 (3)
C3—C4—C5	118.6 (2)	C29—C34—C33	119.0 (3)
C3—C4—C7	120.1 (2)	N3—C35—C32	121.2 (2)
C5—C4—C7	121.3 (2)	N3—C36—C37	123.9 (2)
C4—C5—C6	121.4 (3)	N3—C36—C41	117.4 (2)
C1—C6—C5	118.6 (2)	C37—C36—C41	118.6 (2)
N1—C7—C4	121.6 (2)	C36—C37—C38	120.8 (2)
N1—C8—C9	118.5 (2)	C37—C38—C39	121.1 (2)
N1—C8—C13	123.1 (2)	C38—C39—C40	118.3 (2)
C9—C8—C13	118.1 (2)	C38—C39—C42	121.6 (2)
C8—C9—C10	120.1 (2)	C40—C39—C42	120.2 (2)
O1—C9—C8	115.3 (2)	C39—C40—C41	120.9 (2)
O1—C9—C10	124.6 (2)	O3—C41—C36	115.1 (2)
C9—C10—C11	121.5 (2)	O3—C41—C40	124.6 (2)
C10—C11—C12	118.1 (2)	C36—C41—C40	120.3 (2)
C10—C11—C14	120.2 (2)	C39—C42—C43	121.5 (2)
C12—C11—C14	121.7 (2)	C39—C42—C47	120.1 (2)
C11—C12—C13	120.6 (2)	C43—C42—C47	118.4 (2)
C8—C13—C12	121.7 (2)	C42—C43—C44	120.9 (2)
C11—C14—C19	120.5 (2)	C43—C44—C45	121.2 (2)
C15—C14—C19	118.1 (2)	N4—C45—C44	122.9 (2)
C11—C14—C15	121.4 (2)	N4—C45—C46	118.6 (2)
C14—C15—C16	120.4 (2)	C44—C45—C46	118.2 (2)
C15—C16—C17	121.8 (3)	O4—C46—C45	115.3 (2)
N2—C17—C18	118.6 (2)	O4—C46—C47	124.3 (2)
C16—C17—C18	118.0 (2)	C45—C46—C47	120.5 (2)
N2—C17—C16	123.2 (2)	C42—C47—C46	120.9 (2)
C17—C18—C19	120.0 (2)	N4—C48—C49	120.9 (2)
O2—C18—C19	124.8 (2)	C48—C49—C50	120.1 (3)
O2—C18—C17	115.2 (2)	C48—C49—C54	121.3 (3)
C14—C19—C18	121.7 (3)	C50—C49—C54	118.6 (3)
N2—C20—C21	121.4 (3)	C49—C50—C51	120.8 (3)
C20—C21—C22	120.3 (3)	C50—C51—C52	118.8 (4)
C20—C21—C26	121.6 (3)	Cl4—C52—C51	119.4 (3)
C22—C21—C26	118.1 (3)	Cl4—C52—C53	118.9 (3)
C21—C22—C23	120.9 (3)	C51—C52—C53	121.8 (4)
C22—C23—C24	119.3 (3)	C52—C53—C54	118.9 (3)
Cl2—C24—C25	119.5 (2)	C49—C54—C53	121.1 (3)
C23—C24—C25	121.3 (3)	C29—C30—H30	121.00
Cl2—C24—C23	119.2 (3)	C31—C30—H30	121.00
C24—C25—C26	118.9 (3)	C30—C31—H31	119.00
C21—C26—C25	121.5 (3)	C32—C31—H31	119.00
C3—C2—H2	121.00	C32—C33—H33	120.00
C1—C2—H2	121.00	C34—C33—H33	120.00
C4—C3—H3	120.00	C29—C34—H34	121.00
C2—C3—H3	120.00	C33—C34—H34	121.00
C4—C5—H5	119.00	N3—C35—H35	119.00
C6—C5—H5	119.00	C32—C35—H35	119.00
C1—C6—H6	121.00	C36—C37—H37	120.00
C5—C6—H6	121.00	C38—C37—H37	120.00
N1—C7—H7	119.00	C37—C38—H38	120.00
C4—C7—H7	119.00	C39—C38—H38	119.00
C11—C10—H10	119.00	C39—C40—H40	120.00
C9—C10—H10	119.00	C41—C40—H40	120.00
C11—C12—H12	120.00	C42—C43—H43	120.00
C13—C12—H12	120.00	C44—C43—H43	120.00
C12—C13—H13	119.00	C43—C44—H44	119.00
C8—C13—H13	119.00	C45—C44—H44	119.00
C14—C15—H15	120.00	C42—C47—H47	120.00
C16—C15—H15	120.00	C46—C47—H47	120.00
C17—C16—H16	119.00	N4—C48—H48	120.00
C15—C16—H16	119.00	C49—C48—H48	120.00
C14—C19—H19	119.00	C49—C50—H50	120.00
C18—C19—H19	119.00	C51—C50—H50	120.00
N2—C20—H20	119.00	C50—C51—H51	121.00
C21—C20—H20	119.00	C52—C51—H51	121.00
C21—C22—H22	120.00	C52—C53—H53	121.00
C23—C22—H22	120.00	C54—C53—H53	120.00
C24—C23—H23	120.00	C49—C54—H54	119.00
C22—C23—H23	120.00	C53—C54—H54	119.00
C24—C25—H25	121.00	O3—C55—H55A	109.00
C26—C25—H25	121.00	O3—C55—H55B	109.00
C25—C26—H26	119.00	O3—C55—H55C	109.00
C21—C26—H26	119.00	H55A—C55—H55B	109.00
O1—C27—H27B	110.00	H55A—C55—H55C	110.00
O1—C27—H27C	109.00	H55B—C55—H55C	109.00
H27A—C27—H27B	110.00	O4—C56—H56A	109.00
H27A—C27—H27C	109.00	O4—C56—H56B	109.00
O1—C27—H27A	109.00	O4—C56—H56C	109.00
H27B—C27—H27C	109.00	H56A—C56—H56B	110.00
H28A—C28—H28C	109.00	H56A—C56—H56C	109.00
H28B—C28—H28C	109.00	H56B—C56—H56C	110.00
			
C27—O1—C9—C8	177.9 (3)	C26—C21—C22—C23	−0.2 (4)
C27—O1—C9—C10	−1.0 (4)	C20—C21—C22—C23	−178.8 (3)
C28—O2—C18—C17	177.6 (2)	C22—C21—C26—C25	0.9 (4)
C28—O2—C18—C19	−2.5 (4)	C20—C21—C26—C25	179.5 (2)
C55—O3—C41—C40	−0.8 (4)	C21—C22—C23—C24	−0.8 (5)
C55—O3—C41—C36	177.5 (2)	C22—C23—C24—C25	1.1 (4)
C56—O4—C46—C47	0.7 (4)	C22—C23—C24—Cl2	−179.6 (2)
C56—O4—C46—C45	−177.9 (2)	Cl2—C24—C25—C26	−179.7 (2)
C8—N1—C7—C4	−173.7 (2)	C23—C24—C25—C26	−0.4 (4)
C7—N1—C8—C13	45.5 (4)	C24—C25—C26—C21	−0.6 (4)
C7—N1—C8—C9	−141.9 (3)	Cl3—C29—C30—C31	179.4 (3)
C20—N2—C17—C18	138.2 (3)	C34—C29—C30—C31	0.0 (5)
C17—N2—C20—C21	175.3 (2)	Cl3—C29—C34—C33	−178.9 (2)
C20—N2—C17—C16	−48.2 (4)	C30—C29—C34—C33	0.6 (5)
C35—N3—C36—C41	−136.3 (3)	C29—C30—C31—C32	−0.6 (5)
C36—N3—C35—C32	−178.7 (2)	C30—C31—C32—C33	0.6 (4)
C35—N3—C36—C37	47.5 (4)	C30—C31—C32—C35	178.7 (3)
C45—N4—C48—C49	−176.4 (2)	C31—C32—C33—C34	0.0 (4)
C48—N4—C45—C46	−137.8 (3)	C35—C32—C33—C34	−178.1 (3)
C48—N4—C45—C44	48.1 (4)	C31—C32—C35—N3	−177.4 (3)
C2—C1—C6—C5	0.4 (4)	C33—C32—C35—N3	0.7 (4)
Cl1—C1—C2—C3	−179.2 (3)	C32—C33—C34—C29	−0.6 (4)
Cl1—C1—C6—C5	−180.0 (2)	N3—C36—C37—C38	176.7 (3)
C6—C1—C2—C3	0.5 (5)	C41—C36—C37—C38	0.6 (4)
C1—C2—C3—C4	−1.5 (5)	N3—C36—C41—O3	4.6 (4)
C2—C3—C4—C5	1.7 (5)	N3—C36—C41—C40	−177.0 (3)
C2—C3—C4—C7	−178.2 (3)	C37—C36—C41—O3	−179.0 (2)
C3—C4—C5—C6	−0.8 (4)	C37—C36—C41—C40	−0.6 (4)
C5—C4—C7—N1	−3.2 (4)	C36—C37—C38—C39	−0.4 (4)
C7—C4—C5—C6	179.0 (2)	C37—C38—C39—C40	0.4 (4)
C3—C4—C7—N1	176.7 (3)	C37—C38—C39—C42	179.6 (3)
C4—C5—C6—C1	−0.2 (4)	C38—C39—C40—C41	−0.4 (4)
C13—C8—C9—O1	−179.3 (2)	C42—C39—C40—C41	−179.7 (3)
N1—C8—C9—O1	7.7 (4)	C38—C39—C42—C43	−142.5 (3)
N1—C8—C9—C10	−173.3 (3)	C38—C39—C42—C47	38.6 (4)
C9—C8—C13—C12	−0.3 (4)	C40—C39—C42—C43	36.7 (4)
C13—C8—C9—C10	−0.4 (4)	C40—C39—C42—C47	−142.2 (3)
N1—C8—C13—C12	172.3 (3)	C39—C40—C41—O3	178.8 (2)
C8—C9—C10—C11	1.0 (4)	C39—C40—C41—C36	0.6 (4)
O1—C9—C10—C11	179.8 (3)	C39—C42—C43—C44	−179.8 (2)
C9—C10—C11—C12	−0.9 (4)	C47—C42—C43—C44	−0.9 (4)
C9—C10—C11—C14	178.5 (3)	C39—C42—C47—C46	178.2 (2)
C10—C11—C12—C13	0.3 (4)	C43—C42—C47—C46	−0.8 (4)
C10—C11—C14—C15	−34.9 (4)	C42—C43—C44—C45	1.7 (4)
C10—C11—C14—C19	143.5 (3)	C43—C44—C45—N4	173.4 (2)
C12—C11—C14—C15	144.5 (3)	C43—C44—C45—C46	−0.8 (4)
C12—C11—C14—C19	−37.1 (4)	N4—C45—C46—O4	3.4 (3)
C14—C11—C12—C13	−179.2 (3)	N4—C45—C46—C47	−175.3 (2)
C11—C12—C13—C8	0.4 (4)	C44—C45—C46—O4	177.9 (2)
C11—C14—C15—C16	178.6 (2)	C44—C45—C46—C47	−0.8 (4)
C15—C14—C19—C18	1.1 (4)	O4—C46—C47—C42	−176.9 (2)
C11—C14—C19—C18	−177.4 (2)	C45—C46—C47—C42	1.6 (4)
C19—C14—C15—C16	0.2 (4)	N4—C48—C49—C50	−160.4 (3)
C14—C15—C16—C17	−1.2 (4)	N4—C48—C49—C54	18.7 (4)
C15—C16—C17—C18	0.9 (4)	C48—C49—C50—C51	177.2 (3)
C15—C16—C17—N2	−172.7 (2)	C54—C49—C50—C51	−2.0 (5)
N2—C17—C18—O2	−5.7 (3)	C48—C49—C54—C53	−178.2 (3)
N2—C17—C18—C19	174.3 (2)	C50—C49—C54—C53	0.9 (4)
C16—C17—C18—O2	−179.7 (2)	C49—C50—C51—C52	1.3 (5)
C16—C17—C18—C19	0.4 (4)	C50—C51—C52—Cl4	−180.0 (3)
C17—C18—C19—C14	−1.4 (4)	C50—C51—C52—C53	0.5 (6)
O2—C18—C19—C14	178.7 (2)	Cl4—C52—C53—C54	178.9 (3)
N2—C20—C21—C22	164.9 (3)	C51—C52—C53—C54	−1.5 (5)
N2—C20—C21—C26	−13.7 (4)	C52—C53—C54—C49	0.8 (5)

Symmetry codes: (i) *x*−1, *y*+1, *z*−1; (ii) −*x*−1, −*y*+2, −*z*.

### Hydrogen-bond geometry (Å, °)

**Table:**

Cg2, Cg5 and Cg7 are the centroids of the C8–C13, C29–C34 and C42–C47 rings, respectively.

**Table:**

*D*—H···*A*	*D*—H	H···*A*	*D*···*A*	*D*—H···*A*
C23—H23···O4^iii^	0.95	2.48	3.348 (4)	152
C31—H31···N1^iv^	0.95	2.57	3.498 (4)	166
C3—H3···Cg5^v^	0.95	2.75	3.642 (3)	156
C30—H30···Cg2^iv^	0.95	2.90	3.641 (3)	136
C33—H33···Cg7^vi^	0.95	2.97	3.850 (3)	154

Symmetry codes: (iii) *x*+1, *y*−1, *z*; (iv) *x*, *y*, *z*+1; (v) *x*−1, *y*, *z*−1; (vi) −*x*+1, −*y*+1, −*z*+1.
